# Identification of New Therapeutic Targets by Genome-Wide Analysis of Gene Expression in the Ipsilateral Cortex of Aged Rats after Stroke

**DOI:** 10.1371/journal.pone.0050985

**Published:** 2012-12-12

**Authors:** Ana-Maria Buga, Claus Jürgen Scholz, Senthil Kumar, James G. Herndon, Dragos Alexandru, Gabriel Radu Cojocaru, Thomas Dandekar, Aurel Popa-Wagner

**Affiliations:** 1 Department of Psychiatry, University of Medicine, Rostock, Germany; 2 Department of Functional Sciences, University of Medicine, Craiova, Romania; 3 Interdisciplinary Center for Clinical Research, Lab for Microarray Applications, University of Würzburg, Würzburg, Germany; 4 Department of Biomedical Sciences, College of Veterinary Medicine, Ames, Iowa, United States of America; 5 Yerkes National Primate Research Center of Emory University, Atlanta, Georgia, United States of America; 6 Department of Bioinformatics, Biocenter Am Hubland, Würzburg, Germany; Charité-Universitätsmedizin Berlin, Germany

## Abstract

**Background:**

Because most human stroke victims are elderly, studies of experimental stroke in the aged rather than the young rat model may be optimal for identifying clinically relevant cellular responses, as well for pinpointing beneficial interventions.

**Methodology/Principal Findings:**

We employed the Affymetrix platform to analyze the whole-gene transcriptome following temporary ligation of the middle cerebral artery in aged and young rats. The correspondence, heat map, and dendrogram analyses independently suggest a differential, age-group-specific behaviour of major gene clusters after stroke. Overall, the pattern of gene expression strongly suggests that the response of the aged rat brain is qualitatively rather than quantitatively different from the young, i.e. the total number of regulated genes is comparable in the two age groups, but the aged rats had great difficulty in mounting a timely response to stroke. Our study indicates that four genes related to neuropathic syndrome, stress, anxiety disorders and depression (*Acvr1c*, *Cort*, *Htr2b* and *Pnoc*) may have impaired response to stroke in aged rats. New therapeutic options in aged rats may also include *Calcrl*, *Cyp11b1, Prcp, Cebpa*, *Cfd, Gpnmb*, *Fcgr2b, Fcgr3a*, *Tnfrsf26, Adam 17* and *Mmp14*. An unexpected target is the enzyme 3-hydroxy-3-methylglutaryl-Coenzyme A synthase 1 in aged rats, a key enzyme in the cholesterol synthesis pathway. Post-stroke axonal growth was compromised in both age groups.

**Conclusion/Significance:**

We suggest that a multi-stage, multimodal treatment in aged animals may be more likely to produce positive results. Such a therapeutic approach should be focused on tissue restoration but should also address other aspects of patient post-stroke therapy such as neuropathic syndrome, stress, anxiety disorders, depression, neurotransmission and blood pressure.

## Introduction

Stroke is a devastating condition afflicting mostly the elderly for which no viable medication exists to improve neurorehabilitation. In particular, great clinical benefit may accrue from deciphering and targeting basic neurobiological mechanisms underlying post-stroke CNS recovery both in structural and functional terms. Studies of stroke in experimental animals have identified a variety of interventions with marked neuroprotective effects, but most of these approaches have failed to benefit aged human stroke victims, perhaps because such therapies have been developed in stroke models using young animals. Indeed, recent studies of experimental stroke in the aged animal reveal age differences that may have more clinical relevance, both for understanding cellular responses to stroke and for identification of beneficial interventions [1]–[3], yet these studies fail to fully explain the better outcome of young rats, possibly because they investigated only a small number of genetic events.

Recent advances in genomics and DNA array technology may lead to a comprehensive insight into the mechanisms underlying differences between aged and young animals in rate and extent of brain repair and regeneration after stroke. Several such studies have employed these techniques with the aim of identifying new therapeutic targets for stroke treatment. Some of these studies revealed changes in transcriptional activity of a variety of genes related to stress response, inflammation, acute- and delayed cell death in young rats [4], [5], while a later study revealed a pathway associated with brain defense and tissue repair in a young mouse model of stroke [6]. An important omission of these studies is that they did not include aged animals. The importance of animal age in the physiological response to stroke is emphasized by a recent study that identified an age-specific sprouting or regeneration transcriptome that differentially regulates the process of brain reorganization after brain infarct in young vs. aged animals [7]. However, the genomic response to stroke is not limited to axonal sprouting, but also includes physiologic, metabolic, apoptotic, immunologic, proliferative, developmental, angiogenic and wound healing processes that are of equal importance to neurological rehabilitation. In a study directed at elucidating the role of some of these additional processes, we employed custom DNA arrays containing genes related to hypoxia signalling, DNA damage and apoptosis, cellular response to injury, axonal damage and re-growth, cell differentiation, dendritogenesis and neurogenesis. We showed an age-related unfolding of genetic events in the contralateral, undamaged hemisphere of post-stroke aged rats, which differed from that seen in young animals [8].

The present study expands and extends this work by taking advantage of recent developments in rat genomics, performing a whole-genome transcriptomic analysis of the perilesional infarct during the acute- and recovery phases following stroke. Further, through data mining and one-by-one gene function search in the context of the pathophysiology of stroke, we assigned 161 newly identified genes to stroke-relevant processes such as those mentioned above.

## Materials and Methods

### Animals

Thirty (30) young (3 to 4 mo of age) and 45 aged (19 to 20 mo) male Sprague-Dawley rats, bred in-house, were used. The two age groups were divided randomly into 3-day and 14 day post-stroke survival groups. In addition, nineteen (19) young (3 to 5 mo of age) and 24 aged (18 to 20 mo) male Sprague-Dawley rats were used as controls. Body weights ranged from 290 to 360 g for the young rats and from 520 to 600 g for the aged rats. The rats were kept in standard cages in a temperature- (22*?*C), humidity- (40–60%), and light period- (07.00–19.00 h) controlled environment and had free access to food and water. All experiments were approved by the University Animal Experimentation Ethics Board as meeting the ethical requirements of the German National Act on the Use of Experimental Animals. All surgery was performed under general anesthesia, and all efforts were made to minimiye suffering.

### Surgery

Cerebral infarction was induced by transcranial interruption of blood flow by transiently lifting the middle cerebral artery with a tungsten hook as previously described [9]. Eighteen hours prior to surgery, rats were deprived of food to minimize variability in ischemic damage that can result from varying plasma glucose levels. Water remained available at all times. The right middle cerebral artery (MCAO) was lifted with a tungsten hook attached to a micromanipulator (Maerzhaeuser Precision Micro-manipulator Systems, Fine Science Tools). Both common carotid arteries were then occluded by tightening pre-positioned thread loops for 90 min. Throughout surgery, anaesthesia was maintained by spontaneous inhalation of 1–1.5% halothane in a mixture of 75% nitrous oxide and 25% oxygen. Body temperature was controlled at 37°C by a Homeothermic Blanket System (Harvard Apparatus). The local changes in blood flow were monitored using a laser Doppler device (Perimed, Stockholm, Sweden), and blood gases were measured at several time points during ischemia. A decrease in the laser Doppler signal to *<*20% of control values was considered to indicate successful MCA occlusion. After 90 minutes, the common carotid arteries were re-opened. Because of the extensive collaterality of the rat brain vasculature, in this stroke model the internal carotides must be closed bilaterally, otherwise there is no stroke. Surgery was performed under antiseptic conditions to minimize the risk of infection. Subsequent to survival times of 3 or 14 days, rats were deeply anesthetized with 2.5% halothane, 75% nitrous oxide and 25% oxygen, and the blood removed by perfusion with neutral buffered saline. Brains were cut into 2 mm slices and the periinfarcted area was microdissected under a microscope and stored at −70°C until use.

### RNA Extraction and RNA Quality Control

After the tissue was homogenized, total RNA was extracted from microdissected tissue using TRIzol reagent (Invitrogen life technologies, Karlsruhe, Germany). Genomic DNA was removed using the Rneasy Plus kit (Qiagen).

### Microarray Hybridization

Prior to sample preprocessing, the integrity of RNA pools was assessed with the RNA 6000 nano kit using the Bioanalyzer 2100 instrument (Agilent, Böblingen, Germany). RNA integrity numbers ranged between 6.5 and 8.2. 200 ng of each sample were processed with the whole transcript (WT) expression kit (Ambion, Darmstadt, Germany), i.e. subjected to RNA amplification via reverse transcription to double-stranded cDNA and subsequent *in vitro* transcription; this was followed by another round of reverse transcription yielding single stranded DNA in sense orientation. Hybridization cocktails were produced after fragmentation and biotin labeling of target DNAs following the protocol of the GeneChip WT terminal labeling kit (Affymetrix, Santa Clara, CA). Microarray hybridization to GeneChip Rat Gene 1.0 ST arrays (Affymetrix) was performed according to the manufacturer’s protocol using the Fluidics Station 450 with the program FS450_0007. CEL files from scanned microarrays were produced with the expression console (Affymetrix).

### Microarray Evaluation

Constantly high quality of microarray data was ensured by visual inspection of scanned images for hybridization artifacts and correspondence analysis of raw and normalized microarray data. Normalizations were performed with the Quantiles method [10], background correction and probe set summary were achieved with Robust Microarray Average (RMA) [11]. Differentially expressed genes were determined for 3 days post-stroke vs. naïve and 14 days post-stroke vs. naïve comparisons. These comparisons were done separately for young and aged animals. The False Discovery Rate (FDR) of differential expression for the described comparisons was estimated with an empirical Bayes methodology employing a lognormal normal data modeling [12]. All analyses were performed in R version 2.14.0 (www.r-project.org) along with Bioconductor (www.bioconductor.org) packages affy, EBarrays and made4.

Each array reflected the expression of 19–24 pooled animal samples. This drastically reduces gene expression variance that is otherwise observed between individually hybridized animal samples. Hence, the power loss due to the smaller array sample size is at least partly compensated for.

### Quantitative Real-time PCR

For qualitative real time PCR (qPCR), we synthesized cDNA from large pools (n = 19−24) of total RNA with the High-Capacity cDNA reverse transcription kit (Applied Biosystems, USA). The qPCR was performed in 96-well 0.1-ml thin-wall PCR plates (Applied Biosystems) in the Step One Plus System (Applied Biosystems). Each 20 µl reaction contained 10 µl iQ SYBR Green Master Mix (BioRad Laboratories, Hercules, CA), 2 µl gene-specific forward and reverse primer mix (Qiagen, Alameda, CA) and 8 µl pre-diluted cDNA. No template controls contained nuclease-free water instead. The cycling conditions were 3 min 95°C to activate iTaq DNA polymerase followed by 45 cycles with 30 s denaturation at 95°C, 30 s annealing at 58°C and 30 s elongation at 72°C. At the end of amplification cycles, melting curves were used to validate PCR product specificity. All samples were amplified in triplicates. Data were analyzed using the ΔΔCt method [13]. The expression levels of genes of interest were normalized to the average of expression level of the two housekeeping genes (Hypoxanthine guanine phosphoribosyltransferase 1, HPRT1 and Ribosomal protein 19, RPL 19) from the same sample. So the relative expression for a gene of interest was defined as the ratio of expression of the gene to that of the housekeeping gene. The fold change for a gene of interest was defined as the ratio of the relative expression in the ipsilateral hemisphere (stroke lesioned, peri infarcted or PI) to that in the naïve animals. All primers have been provided by Eurofinn, Germany.

## Results

**Figure 1:**
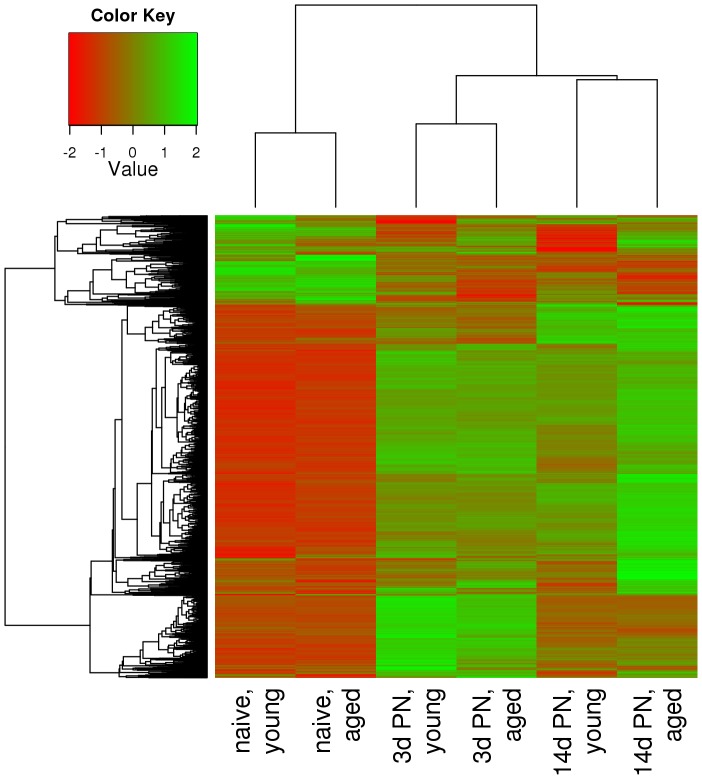
Heatmap of genes differentially expressed between post-stroke and naïve animals. Scaled expression values of all 1,658 differentially expressed genes are shown for each group with light red being the lowest and light green the highest expression level. The depicted dendrograms cluster samples (top) and genes (left) employing average agglomeration and euclidian distance measure.

After raw data normalization and probe set summary, we employed empirical Baysian methodology to analyse expression values of 28,826 transcript clusters for differential expression between post-stroke samples of young and aged rats and their respective controls. This revealed in total 1,658 differentially expressed genes with a two-fold or greater change (up or down) of the transcription rate. Intensities of differentially expressed probe sets from all samples were subjected to agglomerative hierarchical clustering (AHC) and results were displayed as a heat map. The dendrogram shows that relative expression values clearly distinguish naïve rats from their post-stroke littermates (Figure 1). Within the group of infarcted rats, samples cluster according to the time following experimentally induced MCA occlusion. The left-hand dendrogram subdivides expression levels into two major groups; the larger one consists of those probe sets whose expression is generally higher in post-stroke animals than in naïve ones, whereas the smaller group contains transcript clusters with reduced expression. In addition to these major is a group of with a less consistent pattern of expression: they are weakly expressed in untreated animals, strongly expressed 3 days after stroke, and are down-regulated after 14 days. Finally, closer inspection of the heat map reveals that the age of rats also influences gene expression levels.

**Figure 2:**
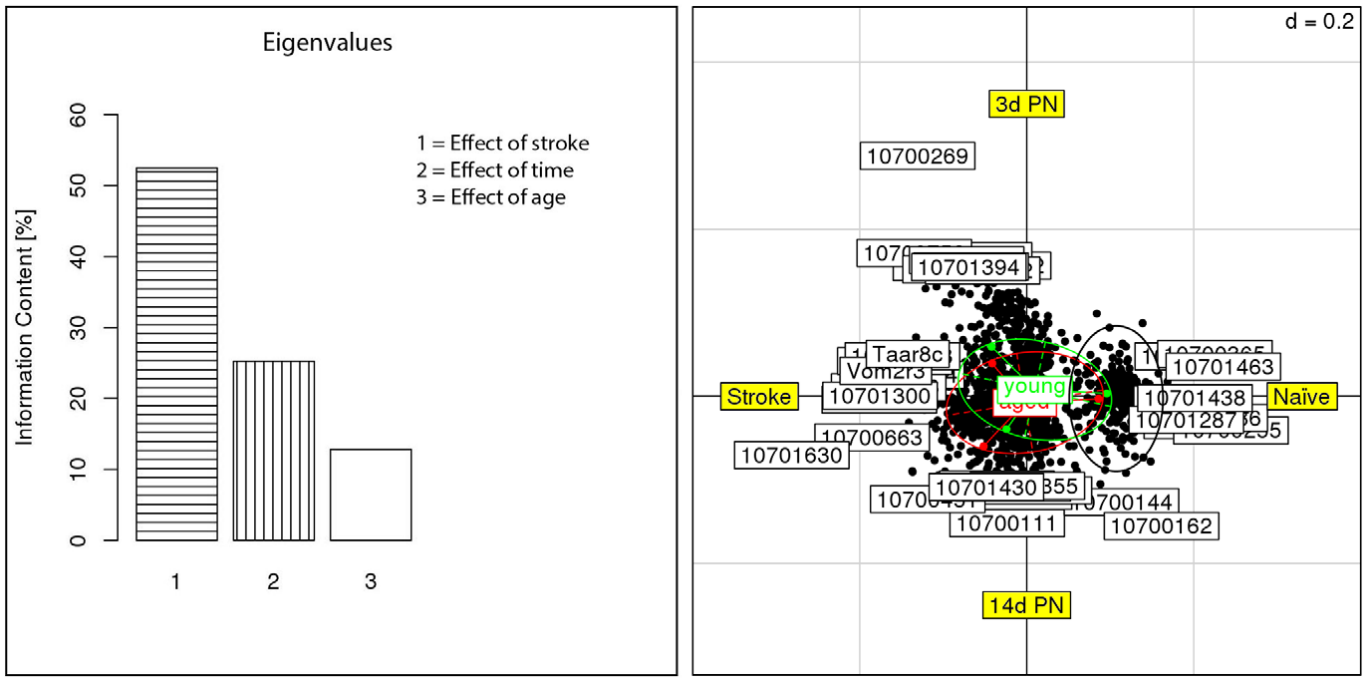
Correspondence analysis of differentially expressed genes and samples grouped by animal age. The left panel depicts the Eigenvalues of the correspondence analysis and shows that the major factors contributing to the variance of stroketomics analysis were stroke (52%), post-stroke time (25%) and age (12%). (Right panel): The first two sources of variability, stroke and post-stroke time formed the coordinates of the right panel. The graph shows the distribution of transcripts (black dotes) as a function of treatment (stroke) and post-stroke time. Samples from young (green) and aged (red) animals particularly differ in their post-stroke response (illustrated by ellipses that form non-parallel planes). Transcripts with characteristic expression in naive samples are encircled in black.

For further examination of the data, we performed a correspondence analysis (COA) for the genes that were differentially expressed according to the Figure 2. By this method, we conclude that the major difference in gene expression between naive and post-stroke samples is produced, as expected, by lesioning. This accounts for 52.5% of the information content. Further, the post-stroke time accounts for 25.2% of the information content while age accounts for 12% (Fig. 2, left panel). On the right panel, the effect of lesioning is given as Axis 1 and the effect of the post-stroke time is given as Axis 2. In this representation we can clearly distinguish between a major cluster of genes assigned to naive animals (black circle) but also the age effect shown by the green circle (for young) and red circle (for aged animals).

### Age-specific Regulation of Gene Expression after Stroke

#### Venn diagrams

**Figure 3:**
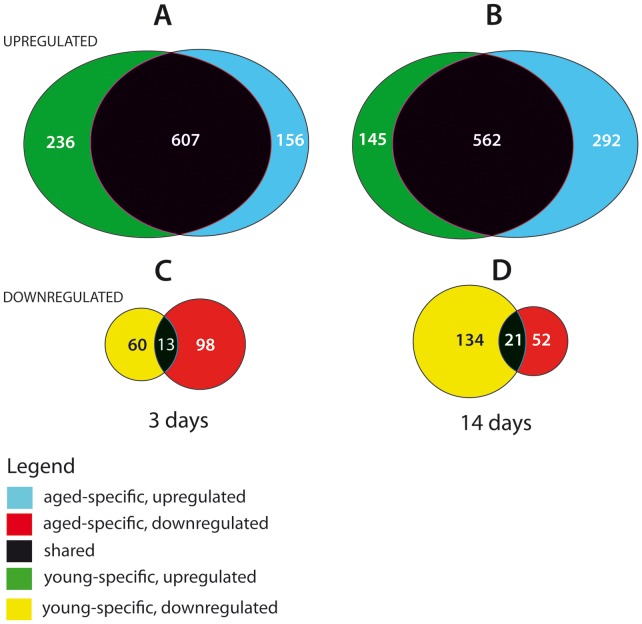
Venn diagrams for 3d and 14d post-stroke showing genes that were up- or down-regulated exclusively in old or young rats, or in both age groups. Note that at 14 days post-stroke, the differences between the age groups were more pronounced.

At day 3 post-stroke, we observed changes in 916 transcript-specific probe sets in young rats, indicating changes in the expression of the corresponding genes. Of these, 218 displayed age-specific upregulation and 52 showed decreased age-specific down regulation. By day 14 post-stroke, fewer probe sets show changes (n = 862), with 138 indicating increased expression levels and 115 showing age-specific decreased mRNA levels (Figure 3A–D).

In aged rats, a similar number of genes was found to be differentially expressed on day 3 post-stroke. Of 874 probe sets, 149 were increased only in aged rats, while 79 showed decreased expression. By day 14 post-stroke, the aged rats had clearly a greater number of differentially expressed genes as compared to young rats at the same time point (n = 927) with 266 more genes being upregulated but fewer genes being down regulated than in young animals (n = 43; 46%) (Figure 3A–D).

Most importantly, the Venn diagrams showed the divergent age-related gene expression with increasing time, ie. the number of upregulated genes in young animals decreased by 39% while the number of downregulated genes increased by 223%. The aged rats showed a mirrored gene expression, ie the number of upregulated genes increased by 187% while the number of downregulated genes *decreased* by 47% (Figure 3 A–D).

### Patterns of Gene Expression after Stroke

**Figure 4:**
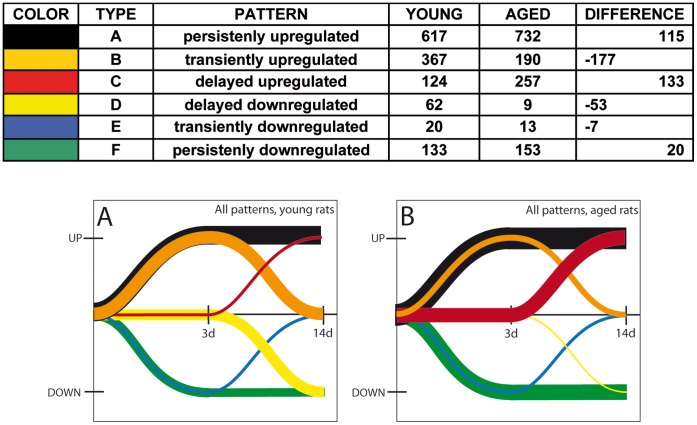
Patterns of gene expression after stroke. There were several distinct patterns of gene regulation: persistently upregulated (black line), transiently upregulated, (orange line), “late-upregulated” (red line), “late-downregulated” (yellow line), transiently downregulated (blue line), and persistently downregulated (green line). Aged animals showed larger numbers than young of genes that were late-upregulated, persistently upregulated and persistently downregulated. The young rats, in contrast, had a much larger number of transiently upregulated and delayed downregulated genes. Note that this representation does not take into account the fold changes for individual genes but the relative change in gene expression at days 3 and 14 post-stroke.

The kinetics of gene expression over a longer time period gives us clues as to what processes in the long run could be defective at the level of transcription in aged rodents. We distinguished several different patterns of gene regulation, as depicted schematically in Figure 4: persistently upregulated (type A, with expression increased both at day 3 and day 14 post-stroke); transiently upregulated, (type B, expression recovered after the acute phase); “late-upregulated” (type C, expression went up after the acute phase); “late-downregulated” (type D, expression went down after the acute phase); transiently downregulated (type E, expression recovered after the acute phase); and persistently downregulated (type F, expression was down both at day 3 and day 14 post-stroke).

A comparison of the global pattern of gene indicated that aged animals showed larger numbers than young of genes that were late-upregulated (133 genes), persistently upregulated (115 genes) and persistently downregulated (20 genes). The young rats, in contrast, had a much larger number of transiently upregulated (177 genes) and delayed downregulated genes (62 vs 9) (Fig. 4, Table 1; A vs B).

**Table 1:** Gene expression for several new potential therapeutic targets to improve post-stroke recovery in aged animals.

Gene Symbol	Function	Drug availability	Y/3d	Y/14d	A/3d	A/d 14d
*Adam17*	post-stroke inflammation	inhibitor available [74]	**↑**	**↔**	**↑**	**↑**
*Atp2b2*	post-stroke excitotoxicity	agonist not available	**↓**	**↓**	**↓**	**↓**
*Cacna2d1*	calcium-channel disregulation	agonist not available	**↔**	**↑**	**↔**	**↓**
*Cacng2*	calcium-channel disregulation	agonist not available	**↓**	**↓**	**↓**	**↓**
*Calcb*	neuropathic syndrome; anxiety; depression	drugs available [29]	**↑**	**↔**	**↓**	**↔**
*Calcrl*	post-stroke blood pressure control	drugs available [39]	**↑**	**↑**	**↑**	**↑**
*Cd36*	limits inflammatory reaction	drugs available [75]	**↑**	**↔**	**↑**	**↑**
*Cd8a*	limits inflammatory reaction	drug available [53], [76]	**↑**	**↑**	**↑**	**↑**
*Cebpa*	post-stroke fibrosis	drug available [52]	**↔**	**↔**	**↑**	**↑**
*Cfd*	limits inflammatory reaction	drugs available [51]	**↑**	**↑**	**↑**	**↑**
*Chrm3*	post-stroke inhibitory neurotransmission	agonist available	**↔**	**↔**	**↓**	**↓**
*Cort*	pain; sleep homeostasis	agonist available [28]	**↓**	**↓**	**↓**	**↓**
*Crhbp*	post-stroke anxiety disorders and stress	under development [35]	**↔**	**↓**	**↓**	**↓**
*Crhr1*	post-stroke anxiety disorders and stress	under development [35]	**↓**	**↔**	**↔**	**↓**
*Cyp11b1*	post-stroke blood pressure control	drugs available [35]	**↓**	**↓**	**↓**	**↓**
*Cyp26b1*	blood vessel morphogenesis	drugs available [62]	**↔**	**↑**	**↑**	**↑**
*Gpnmb*	post-stroke inflammation	drugs available [50]	**↑**	**↑**	**↑**	**↑**
*Htr2b*	neuropathic syndrome; anxiety; depression	drugs available [77], [78]	**↑**	**↑**	**↑**	**↑**
*Kcnk6*	calcium retrieval disregulation	agonist available [77]	**↑**	**↑**	**↑**	**↑**
*Mgp*	extracellular matrix component	drugs available [56]	**↑**	**↑**	**↑**	**↑**
*Mmp8*	tissue remodeling	drug available [40], [54]	**↑**	**↑**	**↑**	**↑**
*Mmp14*	tissue remodeling	drug available [79]	**↑**	**↑**	**↑**	**↑**
*Nr4a1*	neuroprotection	modulator available [80]	**↓**	**↔**	**↓**	**↓**
*Pnoc*	neuropathic syndrome; anxiety; depression	agonists available [27]	**↔**	**↔**	**↔**	**↓**
*Prcp*	blood pressure control	drugs available [40]	**↑**	**↑**	**↑**	**↑**
*Scn7a*	sodium homeostasis	modulators available [24]	**↑**	**↑**	**↑**	**↑**
*Tpcn2*	calcium retrieval disregulation	modulators available [23]	**↑**	**↑**	**↑**	**↑**
*Trim9*	neuroprotection	modulator available [81]	**↓**	**↔**	**↔**	**↓**

Legend

↔ no changes vs contralateral side.

↑ upregulated vs contralateral side.

↓ downregulated vs contralateral side.

y/3d Young, 3d post-stroke.

y/14d Young, 14d post-stroke.

A/3d Aged, 3d post-stroke.

A/14d Aged, 14d post-stroke.

Confirmation of arrays data for new stroke-related genes was done by RT-PCR. Modulation of gene/protein activity by the indicated drugs may, in combination or alone, improve post-stroke recovery in aged animals. For most of the upregulated genes there is a therapeutic option but not for downregulated genes.

Based on known biological functions, as indicated by GOs, we grouped into nine major stroke-relevant processes: “*CNS physiology & homeostasis*”; “*Apoptosis & cell death*”; “*DNA damage & oxidative stress*”; “*Metabolism & cellular energy*”; “*Immune & inflammatory response*”; “*Wound healing & scar formation*”; “*Angiogenesis& vascular remodelling*”; “*Embryonic development & CNS remodelling*”; “*Neurogenesis & synaptic plasticity*”.

**Figure 5:**
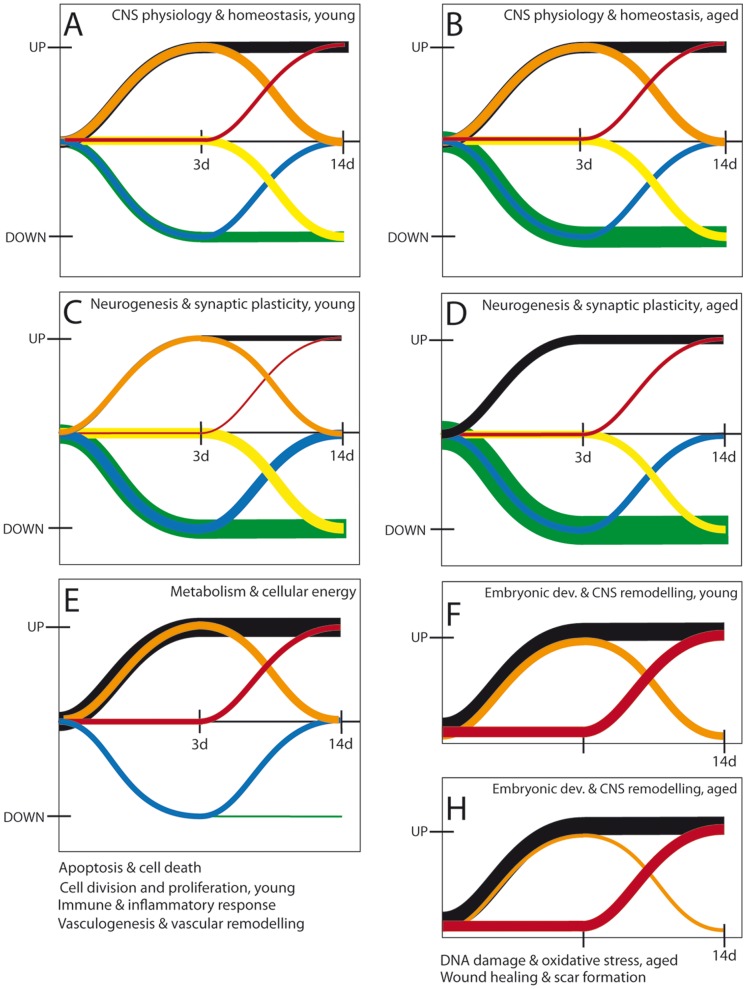
Patterns of gene expression for stroke-relevant processes. Most classes of these new genes were upregulated, with the exception of “*CNS physiology & homeostasis*” and “*Neurogenesis & synaptic plasticity*” which also displayed a large number of downregulated genes.

Most of the stroke-related genes (39%) that were involved in “*CNS physiology & homeostasis”* of young rats displayed upregulation: 19% were persistently upregulated (Type A), 14% were transiently upregulated (Type B), and 6% were upregulated with delay (Type C) (Fig. 5A, B). The number of downregulated genes was slightly higher (43%): 15% were downregulated with delay (Type D) and 11% were transiently downregulated (Type E). Aged rats, however, had twice as many persistently downregulated genes (Type F) as young rats (34% vs. 17%) (Fig. 5A, B). This pattern was found also for genes required for “*Neurogenesis & synaptic plasticity*”. In addition, aged rats had few genes that were transiently up (B-pattern) genes as well as fewer genes that were transiently down (E-pattern) (Fig. 5, C vs D).

The hallmark of the genes involved in “*Embryonic development & CNS remodelling*” was the lack of downregulation in both age groups. Most genes associated with these pathways were persistently upregulated (44 to 55%) followed by transiently upregulated genes (28- to 33%). An age effect was shown by genes upregulated after a delay (12% in young vs 28% in aged rats) (Fig. 5, F vs H). A similar pattern of expression was shown by “*Wound healing & scar formation*” and “*DNA damage & oxidative stress*”. For all other processes the age-dependency of the expression pattern was less evident as illustrated by “*Metabolism & cellular energy*”. A large number of genes in these categories were persistently upregulated (38% in young vs 42% in aged rats). A smaller number (15%) displayed delayed upregulation, or were transiently up-regulated (15%). Fewer still were transiently downregulated (11%) (Fig. 5E).

### Identification of Therapeutic Targets

While the expression pattern gives us clues as to what processes might be de-regulated after stroke in aged animals, it does not yield specific genes, the ultimate goal of stroke genomics. In the following section we make a specific age-related analysis of the genes involved in stroke-relevant processes. RT-PCR was used to verify the target genes.

### CNS Physiology & Homeostasis

#### Upregulated genes common to young and aged

Not surprisingly, the genes displaying persistent upregulation that were shared by the both age groups included some implicated in the pain response to stroke (*Fstl1* and *Htr2b*) [14], blood pressure regulation (*Ace*, *Calcrl*, *Ece1, P2rx4, Prcp*), membrane potential maintenance (*Enpp1* and *Fxyd2*), and excitatory neurotransmission (*Kcnk6, Scn7a*, *Tpcn2*). Of note, *Htr2b* (Serotonin receptor, 5-HT2B) and *Kcnk6* (potassium inwardly-rectifying channel, subfamily K, members 6) were up-regulated 2-fold in aged brains as compared to those of young rats (Table S1).

#### Transiently upregulated genes

A number of genes that were upregulated at day 3 returned to contralateral expression levels by day 14. These included genes implicated in pain (*Calcb*), stress (*Gal, Maoa*) and regulation of blood pressure (*Calca*).

#### Downregulated genes

Aged rats had clearly more downregulated genes at day 14 than young rats. Most of the genes that displayed persistent (Type F) or delayed downregulation (Type D) were involved in responses to pain (*Cort*), stress (*Crhbp, Grm2*), blood pressure regulation (*Chga, Cyb11b1*), membrane potential maintenance (*Atp2b2*), and neurotransmission (*Cacng2*, *Chrm3*, *Chrna4, Gabbr2*). Genes that were transiently downregulated in both age groups included, *Cacng3, Glrb* and *Stx1a* (all neurotransmission). An *age-specific* change in expression was found for genes involved in pain response (*Acvr1c, Pnoc*), stress (*Crhr1*), excitatory neurotransmission (*Grin1*), and inhibitory neurotransmission (*Chrm3*). All of these genes showed delayed- or persistent downregulation in aged, but not in young rats.

Persistent upregulation specific to young rats was displayed by *Lap3* which regulates the cell surface number and stability of alpha3-containing nicotinic acetylcholine receptors. *Htr2a*, a gene heavily implicated in the post-stroke depression and *Kcnj4* (excitatory neurotransmission) were also persistently upregulated in young rats. *Cacna2d1* (excitatory neurotransmission) displayed a mirrored pattern of regulation: it was increased with delay (type C) in young rats, but was downregulated with delay (type D) in the aged animals. All new identified genes and as well as confirmatory genes, ie genes reported to be involved in stroke, are given in Table S1.

### DNA Damage & Oxidative Stress

Strikingly, there were very few genes showing delayed downregulation or persistent downregulation for “*DNA damage & oxidative stress*”. All newly identified genes and three confirmatory genes that showed this pattern are indicated in Table S2.

Genes that were persistently upregulated in both young and aged rats included those associated with oxidative stress (*Atf3*, *Cp*, *Cyp1b1, Mt1a*, *P4hb, Txnip*) and DNA damage (*Brca, Irf1*, *Ripk3*, *Sp100*). Of these, *Atf3* and *Brca1* also displayed a strong age effect, being more strongly upregulated at day 14 in aged than in young rats. *Mt1a* and *Sp100* showed the opposite pattern, with a 2- to 3-fold increased in young as compared to aged rats. Transiently upregulated genes were mostly those associated with DNA repair such as *Nupr1* which was upregulated with delay in both age groups.

### Apoptosis & Cell Death

Apoptosis and cell death are intricately linked to the inflammatory response and to phagocytosis. The vast majority of genes related to these processes were persistently upregulated (86%). All newly identified genes are shown in Table S3.

Upregulated genes included genes whose expression is required for phagocytosis (*Fcgr2b*, *Fcgr3a*), as well several pro-apoptotic genes (*Pycard*, *Ripk3* and *Tnfrsf26)*. Of these, *Fcgr2b*, *Fcgr3a,* and *Tnfrsf26* also displayed a strong age effect, being more strongly upregulated early after stroke in aged (2- to 3-fold) than in young rats. Of note, *Fcgr3a* had a persistently high (3-fold) level of expression in aged animals. Very few anti-apoptotic genes belonging to the classical I-kappaBkinase/NF-kappaBcascade, such as *Bcl2a1d*, were persistently upregulated, especially in aged animals. Likewise, high levels were shown by two prosurvival/antiapoptotic genes (*Serpine1,* 70-fold) and hemeoxygenase 1 (*Hmox1; 50-fold*) at day 3 in young rats as compared to clearly smaller (14 to 17-fold) increases in the aged animals. Two other genes involved in prosurvival/antiapoptotic processes were upregulated with delay, *Ddr1* and *Tpp1*. Notably, discoidin domain receptor tyrosine kinase 1 (*Ddr1*) expression was low in aged rats.

### Metabolism & Cellular Energy

Among the genes persistently upregulated were those involved in energy metabolism (*Cndp1*, *Enpp1*), removal of protein aggregates (*A2m*, *Ttr*) and those required for lipid metabolism, membrane trafficking and protein degradation (*Apobec1*, *Psme1*, *Psmb8*). *Ttr (transthyretin)*, a gene required for removal of amyloid aggregates, showed a strong, highly specific and persistent upregulation (8- to 14-fold) in the aged, but not young, rats. A small number (4%) of genes were persistently downregulated, including *Trhde* (thyrotropin-releasing hormone degrading enzyme), a gene implicated in energy homeostasis and body temperature regulation. All newly identified genes and three confirmatory genes in this category are shown in Table S4.

### Immune & Inflammatory Response

Like those involved with apoptosis and cell death, the vast majority of genes associated with these processes were persistently upregulated. All newly identified genes and five confirmatory genes are shown in Table S5.

First, we noted the early upregulation in aged rats of the gene encoding the receptor for the proinflammatory cytokine interleukin-6 (IL-6r). Among those genes showing persistent upregulation we found *Cfd,* complement factor D required for alternative-pathway complement activation, *Cklf1* encoding the chemokine-like factor 1 which is a potent chemoattractant for neutrophils, monocytes and lymphocytes and *Dpp7,* which encodes a peptidase able to cleave chemokines. A number of new stroke-genes related to T- and dendritic cell activation and differentiation including *Cd 8a*, *Tmem176b*, *Unc93b1, Vamp8*, and *Wipf1* also displayed persistent upregulation. Of these, the cytotoxic lymphocyte marker *Cd8a* was kept at high levels in aged rats at day 14 post-stroke. *Vamp8*, which encodes a SNARE protein of the early/late endosomes, was also persistently upregulated, especially in the aged group.

We also observed the early upregulation of non-canonical I-kappaBkinase/NF-kappaB cascade via *Nfkb2* in both age groups. The non-canonical pathway integrates signals from a subset of TNF receptor family members including *Tnfrsf1a* and *Tnfrsf1b*, both known to be involved in the inflammatory responses to stroke. These two genes were persistently upregulated, while *Tnfrsf12a*, involved in the same non-canonical pathway, was transiently upregulated. We also observed the co-regulation of the transcription factor *Hmgb2* with *NF-kappaB* pathway.

Microglia/macrophage-specific responses to stroke included the persistent upregulation of the genes encoding the microglia activating factors *Cebpa, Cebpd,* the mitogenic factor *Runx1* and the *Ctse* protease, required for chemotaxis and cell adhesion in macrophages, in both age groups. Confirmatory genes, i.e. genes previously known to be upregulated in response to stroke, include *Ccl2, Cd40* and *Cd36*. A strong age-specific upregulation of *Cebpa,* the scavenger receptor-encoding *Cd36* and complement factor D-encoding *Cfd*, was seen in the aged rats (2- to 5-fold increases over the young rats on day 14 post-stroke). *Gpnmb,* which encodes the hematopoietic growth factor-inducible neurokinin 1, also known as osteoactivin, was, in contrast, highly expressed in young but not aged rats.

### Wound Healing & Scar Formation

Scar build-up after stroke is a complex process involving recruitment of glial, fibroblast, and immune cells which function to seal the wounded tissue from the surrounding environment by promoting production of a fibrotic scar. The gene pattern for this process is similar to that described above for “*Immune & Inflammatory responses*”, in that there were practically no downregulated genes. All newly identified genes and one confirmatory gene are shown in Table S6.

Most of the genes associated with these processes were involved in cell division & proliferation and were mostly transiently or persistently upregulated in both age groups. Persistently uregulated genes included *Ccnb1*, *Fabp4, Fabp5, Igfbp7*, all involved in cell proliferation. A gene required for cell cycle progression, *Cdkn1c*, was upregulated with delay in both age groups. Several genes required for cell migration, including *Dab2*, *Itgb1* and *Pdpn*, were also persistently upregulated in both age groups. Genes transiently upregulated in both age groups included cell proliferation-associated genes *Ccnb2, Cdkn2c, Cdkn3,* and *Hbegf*, the latter of which is involved in EGFR signalling. An age-specific effect was seen for *Igfbp7* which was decreased by 2.5 fold in aged rats at day 14 post-stroke vs young rats and *Pdpn* (podoplanin) which was in contrast, increased by 2.5 fold increased in aged rats at day 14 post-stroke.

Astrocyte proliferation is a major event associated with wound healing. Genes persistently upregulated in both age groups included those coding for vimentin (*Vim)* known to be involved in astrocyte proliferation after injuries, as well as *Foxm1, Hmgb2, Map3k1* which were transiently upregulated in both age groups.

Transforming growth factor-beta (TGF-beta) signalling mechanisms at the wound site during the acute phase performs functions central to tissue scarring by mediating astrocytes migration, mesenchymal, fibroblasts and macrophage recruitment, extracellular matrix deposition, re-epithelization, and wound contraction [15]. *Tgfbi* and *Tgfb1i4* were described as TGFβ1-induced ECM binding proteins. *Tgfbi* was transiently upregulated in both age groups, while *Tgfb1i4* was among the few genes persistently downregulated in aged rats only. New stroke-genes *involved presumably* in extracellular matrix remodelling included the transiently upregulated carboxipeptidase-encoding *Cpxm1 and* the permanently upregulated epithelial membrane protein 3 (*Emp3*).

One gene involved in collagen processing showed delayed upregulation (*Pcolce*) in aged rats only. *Itga1,* a gene that plays an essential role in the regulation of mesenchymal stem cell proliferation, displayed a transient upregulation in both age groups.

Several genes involved in fibrosis, the second major scarring process, were persistently upregulated (*Col5a3, Mgp, Mmp8*). *Mgp* encodes a matrix Gla protein and was persistently upregulated, especially in aged rats (increased 2.5 fold over the level in young rats).

### Angiogenesis & Vascular Remodelling

Stroke induces a profound remodelling of the brain vasculature involving mostly permanently upregulated genes (60%) and those undergoing delayed upregulation (17%). All newly identified genes in this category and one confirmatory gene are shown in Table S7.

Genes that were persistently upregulated in both age groups included transcripts of the TGF-beta signalling pathway involved in CNS vascular remodelling and angiogenesis including, *Eng, Gpc3, Mmp14, Scpep1, Tgfbr2* the latter being more strongly upregulated in aged (3-fold) than in young rats during the recovery phase of stroke.

Two genes, *Wnt4* and *Wnt5b,* that belong to the second major signalling pathway involved in CNS vascular remodelling and angiogenesis, showed delayed upregulation in both age groups. Other genes upregulated in both age groups were related to formation of new blood vessels in the CNS during development. These included *Col6a3, Col8a1, Cthrc1, Cxcr4, Ets1, Fbln5, Hhex, Pttg1ip* as well as *Scpep1*, a serine carboxypeptidase associated with vascular injury-induced blood vessel remodelling.

Of the genes showing an age effect, we noted the increased expression in aged rats of the matrix-degrading encoding proteases genes *Adam 17,Mmp14* as well as several genes implicated in vascular development such as *Col8a1, Cthrc1 and Nid2.*

We also observed very high levels of expression of the chemokine *Cxcl1* and collagen type IV, alpha2 (*Col4a2*) in young rats.

### Embryonic Development & CNS Remodelling

In recovery from stroke, adult CNS tissue may require re-activation of pathways implicated in development. Aged rats had 50% more genes showing a temporal disregulation of the expression for several genes that are closely associated with brain development including *Aldh1a2, Crabp2, Mafb, Ninj1, Rbp1, and Tubb6*. Of these we noted age-related increases (2- to 3-fold) for several genes from the morphogenic retinoic acid pathway including *Aldh1a2, Crabp2, Cyp26b1, Mafb, and Rbp1* in the lesioned hemisphere of the aged rats. The largest increases have been noted for *Rbp1* gene that encodes a retinol binding protein which is tightly associated with brain development [16].

Several additional genes implicated in ECM and cytoskeleton remodelling during brain development (*Fbln1, Klk6, Ninj1, Tubb6*) were also persistently upregulated. However, *Vcan*, also in this category, was transiently upregulated. Other synaptic remodelling genes were either transiently (*Racgap1*) or permanently *(Rac2)* upregulated in both age groups. Finally, young rats showed increased levels of two neuronal precursors pro-survival genes *Id3* and *Il6ra.* All newly identified genes are shown in Table S8.

### Neurogenesis & Synaptic Plasticity

Neurogenesis is controlled by genes in several important GOs, including neurite development, corticogenesis, axonogenesis, dentritogenesis and glial differentiation. Most of the genes belonging to this group were downregulated on day 14 post-stroke in both age groups. Very few genes, mostly those required for synaptic activity/turnover (*Adap2, Sema6a*, *Sept2, Svil*), were moderately upregulated in both age groups. However, a gene-by-gene analysis revealed many important age-related differences. Downregulated genes related to corticogenesis and synaptic plasticity included *Arc, Fgf9, Neurod1, Neurod2, Nr4a1, Nr4a2, Rasl10b, Rnf39, Sema6a, Sept2, Tpm3, Trim9, and Vamp1*. Many of these (*Fgf9*, *Neurod1, Nr4a1, Nr4a2, Rasl10b, Rassf7, Rnf39*, *Tpm3*, *Trim9*) were either downregulated with delay or were permanently downregulated more markedly in aged rats, a pattern that may contribute to the slower recovery of function with aging.

Genes related to axonal plasticity were either transiently downregulated in both age groups (*Camk2n2*), downregulated with delay in young rats (*Cntn4*), or persistently downregulated in young rats (*Ntng1*). The peculiarity of this category was the high number of genes (40%) that showed regulation in one group but did not change at all in the other such as *Cntn4, Fgf9, Neurod2, Nr4a2, Ntng1, Rasl10b, Rassf7, Tpm3.* All newly identified genes and one confirmatory gene are shown in Table S9.

## Discussion

Clinical trials aimed at improving functional recovery after stroke have uniformly failed. One reason for this may be that very little genetic information is available describing the post-stroke events. By comparative transcriptomics we identified 161 new stroke-related genes some of which may be used as new therapeutic targets that may help stabilize the aged organisms soon after stroke and facilitate tissue and behavioral recovery after 2 weeks post-stroke.

We showed that in the gene expression dendograms naive rats are clearly separated from post-stroke littermates. Furthermore, observed changes are clearly occlusion time-dependent. Most of the changes observed are active responses, only a smaller group shows inhibitory responses and reduced expression. The gene expression data overview further suggests a specific group of genes with increased expression (compared to untreated animals) 3 days after stroke and with reduced expression later (14 days). Age differences in this pattern, were revealed by the heat map. This latter effect is strongly supported by the correspondence analysis. The first two principal coordinates in this analysis, the effect of stroke and the post-stroke time on gene expression in the perilesional cortex together explained 77% of the variance of stroketomics in aged rats and clearly separate young and older animals. These results (together with the specific annotation and function of the genes) support our notion of an active response to the stroke event at day 3 in the young rats balanced by an inhibitory effect on a roughly similar number of genes (about 1,400) which decays over time (so that at day 14 mostly decreased mRNA levels are seen). However, the aged rats show a set of up-regulated set of genes not observed in the young rats at both 3 and 14 days post-stroke. These genes are involved in wound healing, scar formation, vascular remodelling, and angiogenesis (examples discussed below).

The correspondence, heat map and dendrogram analyses thus independently suggest a differential, age-group-specific behaviour of major gene clusters after stroke. Furthermore, key genes identified were independently verified by RT-PCR.

Gene expression in the young and aged rats diverged with increased time following stroke, as reflected by a decreased number of upregulated genes in young rats and an increased number of upregulated genes in aged rats. A similar pattern of age-group divergence was seen in the downregulated genes, with young animals returning closer to control levels while aged rats showed a greater number of genes with persistently decreased expression. Genes that changed in a similar pattern in both age groups remained the same at both time points.

### Patterns of Gene Regulation

Overall, the pattern of gene expression strongly suggests that the response of the aged rat brain is qualitatively rather than quantitatively different from the young, i.e. the total number of regulated genes is comparable in the two age groups, but the aged rats had great difficulty in mounting a timely response to stroke.

Several changes in gene expression in the lesioned hemisphere were unique to aged animals: (i) a large increase in the number of late upregulated genes; a substantial increase in the number of persistently upregulated genes; (ii) an increase in the number of persistent downregulated genes. The unusually large number of transiently regulated genes in the lesioned hemisphere of young suggests the inability of aged rats to mount a controlled transcriptional response for these genes. Specifically, a large number of genes involved in “*CNS physiology & homeostasis*” were persistently downregulated. While many genes implicated in “*Neurogenesis & synaptic plasticity*” were persistently downregulated, the peculiarity of this category was the high number of genes that showed regulation in one group but did not change at all in the other, suggesting an effect of aging on brain plasticity.

“*Embryonic development & CNS remodelling*” was characterized by a significant increase in the number of late upregulated genes in aged animals and the virtual absence of transiently downregulated genes in both age groups.

### Identification of New Therapeutic Targets for Stroke

In the present work we carried out a genome-wide, transcriptome analysis of post-stroke gene expression in young and aged animals and identified 161 new genes that are of interest to stroke research, as well as some that may be targets for development of stroke therapies (summarized in Table 1). However, since we did not measure the protein level, the term “therapeutic target” is at the time being hypothetical. In the following section we discuss specific age-related characteristics of the genes we have newly identified as having importance in the response to stroke and outline their relevance for behavioural and tissue recuperation after stroke.

Since the young rats recover unexpectedly well in terms of function but do not fully recover at tissue level after stroke, we expect the comparative analysis of gene expression will identify new therapeutic targets for stroke in general, as well as genes that could be therapeutic targets in aged subjects in particular. We distinguish two major approaches for therapeutic intervention, (i) to stabilize the infarct size during the early phase of stroke by controlling inflammation and apoptosis and, (ii) to enhance to repair the capacity of the CNS during the rehabilitation phase that starts at the end of the second week post-stroke on a background of stabilized physiology and system homeostasis. Because young rats recover behaviourally within one week after stroke, we assume that the gene expression is optimal in this age group. In selecting genes as therapeutic targets, preference should be given to those genes for which activity-modulating drugs are already available.

*CNS physiology & homeostasis*-related genes were characterized mainly by the increased number of late downregulated neurotransmission-related genes in aged rats. Post-stroke neurotransmission is strongly affected in the periinfarcted area. This effect is mainly due to persistent downregulation of two genes encoding calcium channels, *Cacna2d1* and *Cacng2*, and to strong upregulation of genes encoding for ion channels *Kcnk6, Scn7a* and *Tpcn2*. *Tpcn2* is important for calcium retrieval from acidic lysosomes [17], [18]. Highest downregulation was seen for *Cacna2d1*, a gene that has been associated with schizophrenia [19]. *Cacng2* is required for compartment-specific AMPA receptor trafficking and synaptic plasticity [20] and mice with mutations in *Cacng2*, display generalised cortical spike-wave discharges [21]. For both *Kcnk6* and *Tpcn2* activity modulators are available [22], [23]. The *Scn7a* gene encodes an atypical sodium channel which is involved in hydromineral homeostasis and osmoregulation. Pharmacological activity modulators of this gene are available [24].

Further, the gene encoding CHRM3 (cholinergic receptor, muscarinic 3) was specifically and persistently downregulated in the aged rat brains. Because of its recent implication in atrial fibrillation/arrhythmia [25] and the availability of an agonist (carbachol), this gene is a good candidate as a therapeutic agent to improve inhibitory neurotransmission in post-stroke rats.

Neglected aspects of patient post-stroke life quality are neuropathic syndrome, stress, anxiety disorders and depression. Our study indicates that four genes related to these processes may have impaired response to stroke in aged rats. These are activin A receptor, type IC (*Acvr1c*) expression along with cortistatin (*Cort*), serotonin receptor 2b (*Htr2b*) and preprononiceptin (*Pnoc*). These genes are required for homeostatic mechanisms of general brain activity including stress, anxiety disorders, depression and sleep behaviour in adults. Of these *Cort*, which is also involved in sleep homeostasis [26] remained downregulated on day14 post-stroke in both age groups, and *Acvr1c, Htr2b* and *Fstl1* all involved in pain response, remained high at day14 in aged rats. Drugs that influence the activity of *Acvr1c* and *Htr2b* genes are available [27]–[32].

The gene coding for the neuropeptide corticotropin-releasing hormone (*Crh*) and the gene encoding its receptor type 1 (*Crhr1*) are key regulators of the neuroendocrine stress axis [33]. One new gene that is a target of the neuroendocrine stress axis is *Crhbp* [34]. *Crhr1* and *Crhbp* were either transiently or persistently downregulated both in young and aged rats in response to stroke and, more importantly, their expression did not recover by day14 post-stroke, remaining especially low in aged animals. Both agonists and antagonists for these pathways are under development [35].

Our results strongly indicate a disregulation of post-stroke blood pressure, a condition often neglected in clinical settings. New therapeutic options suggested by our study for control of blood pressure include the genes *Calcrl*, *Cyp11b1* and *Prcp* [36]. CALCRL (calcitonin receptor-like) is a multifunctional vasodilator peptide that was persistently upregulated in aged rats whereas *Cyp11b1* is associated with essential hypertension and had decreased levels in both age groups [37]. Activity modulators available for all three of these genes [38]–[40].

Perturbations in calcium balance, have received attention as major cause of axonal damage and neuronal dysfunction in many pathological conditions [41]. Ca^2+^-ATPase-encoding *Atp2b2* plays a major role in clearing Ca^2+^ from the neuronal cytoplasm and was downregulated after stroke. Since a wealth of activators and inhibitors exist for this enzyme, it should be investigated as a therapeutic target balance intracellular calcium concentration after stroke.

Oxidative stress and DNA damage-related genes have been well studied in stroke models. However, none of the proposed drugs targeting these genes was efficient in reducing oxidative stress and DNA damage incurred cell death. Therefore further therapeutic targets are needed.

Post-stroke young rats were better protected against oxidative stress by persistently upregulating genes coping with the oxidative stress like the antioxidant zinc-binding proteinmetallothionein 1a (*Mt1a*) and the transcriptional regulator, *Sp100*. The gene encoding the thioredoxin-interacting protein (*Txnip*), an endogenous inhibitor of the anti-oxidant thioredoxin, was upregulated with delay in young animals, suggesting that most of oxidative events may occur during the first week post-stroke. Likewise, young animals had a higher level of expression for three prosurvival/antiapoptotic genes, tissue plasminogen inhibitor (s*erpine1*), hemeoxygenase 1 (*Hmox1*), and discoidin domain receptor tyrosine kinase 1 (*Ddr1*) as compared to aged animals.

Young rats also had better synchronized gene expression as was illustrated by the coordinated co-expression of genotoxic stress-induced genes *Brca1* and *Irf1* at day 3 post-stroke. Aged rats, on the contrary, had early post-stroke increases in expression of phagocytosis-promoting genes like *Fcgr2b* and *Fcgr3a*, and for the pro-apoptotic acting gene, tumor necrosis factor receptor superfamily, member 26 (*Tnfrsf26)*. The binding of IgG immune complexes to Fcgamma receptors 2b and 3a on monocytes triggers potent inflammatory responses leading to exacerbated tissue injury in disease [42], [43]. Potent inhibitors of *Fcgr2b* and have been recently been described for *Fcgr3a* [44], [45]We noted that several genes involved in “*Metabolism & cellular energy”* showed opposite patterns of post-stroke changes in young and aged rats. One of these, alpha-2-macroglobulin, a gene responsible for removal of protein aggregates, was highly expressed in young vs aged rats. Another gene in this class, *transthyretin*(*Ttr*), a gene required for removal of amyloid aggregates, was highly up-regulated in aged rats. These findings suggest that the time course of the formation and degradation of macromolecular aggregates in the two age groups is quite different, confírming our previous studies on precipitous accumulation of amyloid aggregates in the injured hemisphere of aged rats [46], [47]. Intriguingly, the young rats did not upregulate transthyretin at all. Also remarkable was the permanent downregulation of the enzyme3-hydroxy-3-methylglutaryl-Coenzyme A synthase 1 in aged rats, a key enzyme in the cholesterol synthesis pathway for which a number of good inhibitors exist but no activators.

New genes whose modulation may help limit the inflammatory reaction in aged rats may also include *Cebpa*, *Cfd* and *Gpnmb*. GPNMB, (glycoprotein nonmetastatic melanoma protein, also known as osteoactivin) is induced in macrophages by IFN-gamma and lipopolysaccharide and acts as a feedback regulator of proinflammatory responses. This gene was also persistently and strongly upregulated in both age groups, and is amenable to drug manipulation [48]–[50]. Another interesting therapeutic candidate is the pro-inflammatory adipokine gene *Cfd* which encodes a member of the serine protease family and for which a number of inhibitors exist [51]. Finally, the microglia-activating factor (*Cebpa*), is amenable to pharmacological manipulation by G-CSF [52], a pleiotropic drug that was shown to improve recuperation in aged, post-stroke rats by our group [53].

Scar build-up and post-stroke fibrosis are believed to create an obstacle toward axonal re-growth after stroke. Therefore other potential therapeutic target includes gene products that contribute to the post-stroke fibrosis and that were also strongly upregulated including *Mmp8* and *Mgp*. Neutrophil collagenase (*Mmp8*) can be inhibited by clodronate [54]. *Mgp* (matrix Gla protein) is a gene coding for an extracellular matrix protein and is implicated in wound healing [55]. *Mgp* expression can be modulated by drugs such as dexamethasone [56].

Both scar build-up and fibrosis are controlled mainly by astrocytes, microglia and fibroblasts and are intimately associated with genes required for cell proliferation. Genes of this type were frequently upregulated, either transiently or persistently, in both age groups. However, in order to be able to define further therapeutic targets, further studies are required to unequivocally identify the cellular specificity of these genes.

Genes involved in *angiogenesis & vascular remodelling* appeared at first sight to be largely unaffected by age, as most were regulated in similar patterns in the two age groups. However, one third of the genes were increased with suboptimal timing in aged rats, either on day 3 post-stroke (*Adam17*, *Gpc3, Mmp14, Nid2, Tagln, Wnt5b*) or on day 14 after stroke (*Col4a2, Col8a1, Cthrc1*, *Cxcl1, Gpc3, Tgfbr2*). Of the genes that increased at the day3 time point, we noted the increased expression of the matrix-degrading encoding proteases genes *Adam 17* and *Mmp14*. It is conceivable that excessively high activity of matrix-degrading proteases may diminish angiogenesis, a prerequisite for tissue regeneration. Therefore inhibiting *Adam 17* and *Mmp14* geneproducts by available compounds [57], [58] may increase the chance of increased angiogenesis and hence a better tissue restoration in aged brains after stroke.

Young rats on the other hand had very high levels of expression of the chemokine *Cxcl1* and basement membrane component, collagen type IV, alpha2 (*Col4a2*) in young rats suggesting that these two genes are beneficial for efficient angiogenesis whereas basement membrane components encoded by *Col8a1*, *Cthrc1* and laminin-binding protein nidogen 2 (*Nid2),* though necessary for blood vessel formation, are upregulated with delay in aged rats; this delay may disrupt angiogenesis in this age group.

Following stroke most of the developmental pathways are activated in both age groups. Identification of gene networks that become active early after stroke may help us to illuminate basic genetic mechanisms underlying post-stroke at the early stages of tissue recovery after stroke. It may also promote design of better pharmacological approaches to sustain developmental pathways that are do initiated shortly after stroke. Aged rats had 50% more genes showing both a temporal disregulation and surprisingly an increased gene expression related to brain development including *Aldh1a2, Crabp2, Cyp26b1, Mafb, Ninj1, Rbp1 and Tubb6.* Of these, the aldehyde dehydrogenase (*Aldh1a2*) is essential for production of the powerful morphogen retinoic acid (RA) production while *Cyp26b1* gene encodes for an enzyme that metabolizes retinoic acid and therefore can be regarded as an antagonist of the *Aldh1a2* gene. Closely related to *Rbp1* expression is the expression of *Aldh1a2, Crapbp2,* and the Hox-regulating genes *kreisler*/*mafB*, two genes implicated in neural development [59]–[60].

Retinoic is a strong morphogen during CNS development [61]. An increased expression of the retinoic acid pathway in aged rats is quite intriguing and may suggest that an overt re-activation of the retinoic acid pathway is undesirable. Therefore, the application of the pharmacological *Cyp26* blocker R115866 [62], may help clarify the role of retinoic acid in post-stroke tissue restoration.

Finally, the more complete tissue recovery in young rats may be due to increased levels of neuronal precursors, pro-survival genes, the helix-loop-helix (HLH) family of transcription factors, *Id3,* and the interleukin receptor 6a (*Il6ra*) [63]–[65].

Post-stroke axonal growth was compromised in both age groups [66]. Key genes associated with axonal growth such as *Contactin 4* (*Cntn4*) and *Netrin-G1* (*Ntng1*), were either persistently downregulated or displayed a delayed pattern of downregulation. Disruption of *Cntn4* is known to cause developmental delay and mental retardation, as well as autism [67], while *Ntng1* mutations are a cause of Rett syndrome and have been implicated in the pathophysiology of schizophrenia [68].

Genes whose expression was persistently upregulated in both age groups were quite rare. Among the genes in this class were *Adap2*, *Sema6a* and *Sept2*. *Adap2* (ArfGAP with dual PH domains 2) is highly expressed in the nervous system with peak expression in early postnatal development [39]. Late upregulation of *Adap2* in the lesioned hemisphere of aged rats confirms the presence of disregulated gene expression in aged animals. *Sema6a* has been implicated in axonal guidance [69] while *Sept2* has been recently shown to be upregulated in neurospheres [70].

Genes that were persistently downregulated in the aged rats were more numerous than in the young group and included a group of genes involved in synaptic activity-induced neuroprotection (*Nr4a1*, *Rnf39*, *Tpm3 and Trim9*). *Nr4a1* and *Tpm3* encode an activity-regulated cytoskeleton-associated protein but most of these genes have also been implicated in synaptic plasticity via ubiquitin-mediated turnover [71]–[73].

Many genes implicated in the *neurogenesis & synaptic* plasticity were temporarily downregulated. We hypothesize that these genes are required for adult brain functionality i.e. they are expressed in the normal brain but are downregulated following damage to the CNS. Eventually their expression recovers upon restoration of brain homeostasis. In this respect the young rats had more genes encoding neuroprotective factors (*Fgf9, Nr4a1, Tpm3*) that had recovered by day14 post-stroke suggesting a better control of adult brain plasticity in young animals.

### Conclusions

To date, all monotherapeutic attempts to prevent or lessen brain damage following stroke have failed. In view of our findings that stroke impacts a wide range of systems in an age-dependent manner, from CNS physiology to CNS regeneration and plasticity, the failure of therapies aimed at only a single target system is perhaps inevitable. Our results suggest that a multi-stage, multimodal treatment in aged animals may be more likely to produce positive results. Such a therapeutic approach should be focused on tissue restoration but should also address other aspects of patient post-stroke therapy such as neuropathic syndrome, stress, anxiety disorders, depression, neurotransmission and blood pressure.

## Supporting Information

Table S1 **CNS physiology and system homeostasis-related genes verified by RT-PCR.**
(XLSX)

Table S2 **DNA damage and oxidative stress-related genes verified by RT-PCR.**
(XLSX)

Table S3 **Apoptosis & cell death-related genes verified by RT-PCR.**
(XLSX)

Table S4 **Metabolism & cellular energy-related genes verified by RT-PCR.**
(XLSX)

Table S5 **Immune and inflammatory response-related genes verified by RT-PCR.**
(XLSX)

Table S6 **Wound healing and scar formation-related genes verified by RT-PCR.**
(XLSX)

Table S7 **Angiogenesis & vascular remodelling-related genes verified by RT-PCR.**
(XLSX)

Table S8 **Embryonic development& CNS remodelling-related genes verified by RT-PCR.**
(XLSX)

Table S9 **Neurogenesis & synaptic plasticity-related genes verified by RT-PCR.**
(XLSX)
